# The effect of Patient’s Own Medication use on patient’s self-reported medication knowledge during hospitalisation: a pre-post intervention study

**DOI:** 10.1186/s12913-022-07752-6

**Published:** 2022-03-30

**Authors:** Loes J. M. van Herpen-Meeuwissen, Bart J. F. van den Bemt, Hieronymus J. Derijks, Patricia M. L. A. van den Bemt, Barbara Maat, Hein A. W. van Onzenoort

**Affiliations:** 1 Department of Pharmacy, Radboud University Medical Centre, Radboud Institute for Health Sciences, Nijmegen, The Netherlands; 2 Department of Pharmacy, Elisabeth-TweeSteden Hospital, Tilburg, The Netherlands; 3 Department of Pharmacy, Sint Maartenskliniek, Nijmegen, The Netherlands; 4 Department of Clinical Pharmacy and Toxicology, Maastricht University Medical Center+, Maastricht, Netherlands; 5 Department of Pharmacy, Jeroen Bosch Hospital, ’s-Hertogenbosch, the Netherlands; 6 Department of Clinical Pharmacy and Pharmacology, University of Groningen, University Medical Center Groningen, Groningen, The Netherlands

## Abstract

**Background:**

Improving patient’s medication knowledge and consequently medication use is essential for optimal treatment outcomes. As patient knowledge about medication is currently suboptimal, interventions to optimise medication knowledge are necessary. Implementation of Patient’s Own Medication (POM) in which patients bring their outpatient medication to the hospital, and nurses administer these during admission, may increase medication knowledge. The aim of this study is to explore the impact of POM use on self-reported medication knowledge of hospitalised patients compared to standard care. Patient’s sense of medication safety, attitude to the provision of information, and to inpatient medication use were studied in both standard care and during POM use too.

**Method:**

In this nationwide intervention study perceived medication knowledge was assessed with a questionnaire pre and post implementing POM use. The questionnaire assessed perceived medication knowledge at admission and discharge, medication safety during hospitalisation, the provision of information during hospitalisation and at discharge, and inpatient medication use during hospitalisation. Patients’ answers were categorised into positive and negative/neutral. The proportion of patients with adequate medication knowledge, in the standard care and POM use group at hospital admission and discharge, were calculated and compared with adjustment for potential confounders.

**Results:**

Among the 731 patients (393 received standard care and 338 POM) who completed the questionnaire (80.2%), POM use seemed to be positively associated with self-reported knowledge on *how* to use medication at discharge (adjusted OR: 3.22 [95% CI 2.01–5.16]). However, for the other two knowledge related statements POM use was not associated. Medication knowledge at admission was the most important variable associated with perceived medication knowledge at discharge. The majority perceived POM use to be safer (52.9% of standard care patients versus 74.0% POM users; *P* <  0.01), POM users knew better which medicines they still used during hospitalisation (85.8% versus 92.3% resp.; *P* = 0.01), and most patients preferred POM use regardless of having experienced it (68.2% versus 82.2% resp.; *P* <  0.01).

**Conclusion:**

POM use positively affects patient’s medication knowledge about *how* to use medication and patients’ perception of medication safety. With POM use more patients have a positive attitude towards the provision of information. The majority of patients prefer POM use. In conclusion, POM use seems a valuable intervention and requires further investigation.

**Supplementary Information:**

The online version contains supplementary material available at 10.1186/s12913-022-07752-6.

## Background

Medication is designed to help diagnose, treat, cure, mitigate or prevent a disease [1]. Inappropriate medication use could compromise this resulting in negative clinical outcomes like avoidable hospital admissions, progression of disease, disease related complications, premature disability, and death, which are detrimental for both the community and the individual patient [2–8]. In addition, inappropriate medication use is associated with increased costs [9]. Improving patient’s medication knowledge is essential in disease management as it positively influences correct medication use and adherence to treatment, and thus attainment of clinical goals contributing to quality of life [10–14].

Medication knowledge includes patient’s ability to recall a drug’s name, indication, dosage regimen, and frequently occurring adverse drug reactions [10–17]. Despite its importance in disease management, it has been estimated that more than 50% of the patients, amongst variety of diseases and nationalities, have an inadequate knowledge of the medication they are taking [10–17]. In the inpatient setting this problem seems even greater, as 96% of the patients cannot name at least one medication used during hospitalisation [18]. At discharge, the consequences of this problem become evident. The majority of patients have limited understanding of the impact of their changed medication regimen (like adaptation of dosage, starting and discontinuing medication) [19]. Therefore, interventions to optimise medication knowledge are necessary.

An opportunity to optimise medication knowledge during hospitalisation is Patient’s Own Medication (POM) use. In this concept, patients bring their outpatient medication to the hospital, and nurses administer this medication during admission. POM use may increase patient’s involvement in pharmacotherapy during hospital stay as patients have the responsibility of bringing their medication to the hospital, and medication remains recognizable during hospitalisation. Consequently, patients may be more in control of their pharmacotherapy. This may positively affect their medication knowledge, but has not been studied yet. Therefore, the aim of this study is to explore the impact of POM use on self-reported medication knowledge of hospitalised patients compared to standard care (SC). Furthermore, patient’s sense of medication safety, attitude to the provision of information, and to inpatient medication use were studied in both SC and during POM use.

## Methods

### Design

We conducted a multicentre nationwide prospective implementation study with a pre-post design. The study was designed to implement POM as standard practice and to have as less impact on daily clinical practice as possible. Therefore, only the most essential data were collected. Consequently, we did not collect data about how many patients were eligible for participation and how many patients participated in the study. The study population consisted of all adult patients admitted to eight medical wards of seven Dutch hospitals. Data were collected during a study period of four months between 2015 and 2017. Of these hospitals three were university, two teaching, one general, and one specialised hospital. The included departments were: Cardiology, Internal medicine, Haematology, Pulmonology, Medical Oncology, Orthopaedics, the combined wards Internal medicine / Gastroenterology / Geriatrics, and Gynaecology / Urology / Otorhinolaryngology. In the pre-implementation phase, patients received SC, thereafter POM use was implemented, and subsequently patients received POM use in the post-implementation phase (both 2 months observation time).

### Pre-intervention: standard care

As part of SC in The Netherlands each patient receives medication reconciliation at hospital admission in order to make an inventory of a patient’s actual medication use (home medication). Based on this and the hospital’s formulary, physicians electronically order medication. Non-formulary medication is often substituted to a different brand and/or a different substance of the same pharmacological subgroup. Medication, mostly unit dose, is distributed from the inpatient pharmacy to the wards, and is administered by nurses. Furthermore, information about started or changed pharmacotherapy is provided to the patient during hospitalisation. During hospitalisation patients are not involved in the medication process. At discharge, substituted medicines are resubstituted to the patient’s original home medication, patients receive an updated medication list, and additional information on started or changed pharmacotherapy is provided. When necessary, patients receive prescriptions for newly started or changed pharmacotherapy which are filled by the outpatient pharmacy.

### Intervention: POM use during hospitalisation

POM use during hospitalisation was described before by the research group [20]. During the intervention period, the patients were asked to bring their own medication, in the original (regularly multi dose) packages, to the hospital. In case of an acute hospitalisation, the patient’s relatives were asked to bring the patient’s medication within 24 h. Medication reconciliation took place as in SC. Thereafter, a physician electronically ordered the patient’s exact home medication without taking the hospital’s formulary into account. Consequently, medication substitution did not take place. POM stock was checked by a nurse or pharmacy technician for completeness, shelf life, quality (by observation), and quantity. Thereafter, the medication was stored per patient. Nurses administered POM. Newly started medication that should be continued at home and POM that ran out of stock were provided in outpatient packages by the pharmacy and stored per patient too. Medication information was given as in SC. At discharge, POM was collected, including newly started therapies, and handed over to the patient. As in SC, patients received an updated medication list.

### Study population

All adult patients admitted to the participating wards during the study period were eligible for participation if they were using medication at home. Only patients using medication that could not be traced back to its original package were excluded, i.e. patients with individualised pre-packaged medication (either by an automated dispensing system or medication organizer box), without the possibility of bringing the original medication package to the hospital.

### Data collection

In both study periods of two months duration, patients were asked to complete one questionnaire with statements on perceived medication knowledge, medication safety, the provision of information, and inpatient medication use (see Additional file 1). Healthcare professionals handed over the questionnaire to all admitted patients on the ward who were eligible to participate. If patients filled in the questionnaire they consented to participate in this study, number of patients who did not (want to) participated were not collected. This questionnaire was designed by the research team based on published literature and their expertise with medication process adoptions and designing patient questionnaires [21, 22]. Thereafter the questionnaire was proofread by patients and further textual refined resulting in the final questionnaire. Patients were asked to respond to six statements at admission (A1–6), six other statements during hospitalisation (B1–6) and five statements at discharge (C1–5). The three statements on medication knowledge were asked twice, at admission (A1–3) and discharge (C1–3) respectively. Patients were asked to score on a 5-point Likert scale (ranging from totally disagree to totally agree), how much they agreed with each statement. Patient characteristics (gender, year of birth, level of education, marital status, native language, and help with medication use at home) were collected as well. Data on current medication use or number of medication changes during hospitalisation were not part of our study.

### Outcome

The main outcome of this study was the proportion of patients with adequate self-reported medication knowledge in the POM use group and the SC group at hospital discharge and the identification of determinants associated with it. Patients’ answers to the questionnaire’s statements were dichotomized into positive (representing answers agree or totally agree) and negative/neutral (representing answers: disagree, totally disagree, neutral, unknown or not applicable). Self-reported medication knowledge was defined adequate if the response was positive per statement on medication knowledge. The secondary outcomes were: patient’s sense of medication safety, attitude to the provision of information, and to inpatient medication use.

### Data analysis

Data were descriptively analysed and reported as counts and percentages (except for the continuous variable age which was reported as mean and standard deviation). No sample-size calculation was performed, as this was an exploratory study. The proportion of patients who reported adequate medication knowledge at admission and discharge was calculated for both study periods. The differences between groups at baseline were analysed per characteristic using Pearson Χ^2^ test (or independent t-test for age). Our primary outcome was analysed in a multivariate logistic full model regression analysis with self-reported medication knowledge at discharge as dependent variable. Possible confounders (like gender) were entered into the model. Self-reported medication knowledge at admission was entered into the model as well to adjust for baseline knowledge. If a confounder was missing for a case listwise deletion was performed. The proportion of patients that were positive towards a statement on medication safety, provision of information, and inpatient medication use during SC and POM use were calculated and compared using Pearson Χ^2^ test. Data were analysed using SPSS (IBM Corp. Release 25.0.0.1. IBM SPSS Statistics for Windows, Version 25.0. Armonk, NY: IBM Corp). Results were assumed to be significant when *P* <  0.05 for all statistical analyses.

## Results

### Study population

In total 731 patients (80.2%) responded to all statements (part A, B and C of the questionnaire). Of these, 393 patients received SC and 338 received POM use. The mean age of patients was 62.4 (SD 14.7) and 61.1 (SD 15.7) years old, in the SC and POM use group respectively (Table 1). Significantly more patients received help with medication management at home and less patients claimed to always take their prescribed medication in the SC group compared to POM users (22.6% versus [vs] 13.6 and 89.3% vs 96.2%, respectively).

**Table 1:** Study population demographic

Demographic characteristics	Standard care *n* = 393		POM use *n* = 338		***P*** value*
Female^a^, n (%)	172	(44.6)	141	(42.0)	0.48
Age^b^ (mean and standard deviation)	62.4	(14.7)	61.1	(15.7)	0.28
Marital status^c^ (n, %)					0.57
- Single	45	(11.5)	46	(13.7)	
- Partner, not married	40	(10.2)	37	(11.0)	
- Married	250	(63.8)	214	(63.9)	
- Divorced	20	(5.1)	18	(5.4)	
- Widow/widower	34	(8.7)	19	(5.7)	
- Unknown	3	(0.8)	1	(0.3)	
Educational level^d^ (n, %)					0.27
- Elementary school	25	(6.4)	25	(7.5)	
- Lower secondary education	141	(36.1)	116	(34.6)	
- Upper secondary school	125	(32.0)	95	(28.4)	
- Bachelor degree or higher	98	(25.1)	92	(27.5)	
- Unknown	2	(0.5)	7	(2.1)	
Native Dutch (n, %)	366	(93.1)	325	(96.2)	0.07
Help with medication management at home^e^ (n, %)	65	(22.6)	45	(13.6)	< 0.01
Adequate self-reported medication use^f^ (n, %):					
01. I always take my prescribed medicine	351	(89.3)	325	(96.2)	< 0.01
02. I always take the prescribed amount of medicine	358	(91.1)	319	(94.4)	0.09
03. I always use my medicine at the prescribed time	336	(85.5)	296	(87.6)	0.41

Used aberration: *POM* Patient’s Own Medication

* Pearson Chi-Square test for categorical data and independent t-test for continuous data

^a^Data of 7 and 2 records were missing

^b^Data of 37 and 31 records were missing

^c^Data of 1 and 3 records were missing

^d^Data of 2 and 3 records were missing

^e^Data of 105 and 6 records were missing

^f^Patients that were positive (agree or totally agree) towards a statement at admission

### Main outcome

**Fig. 1:**
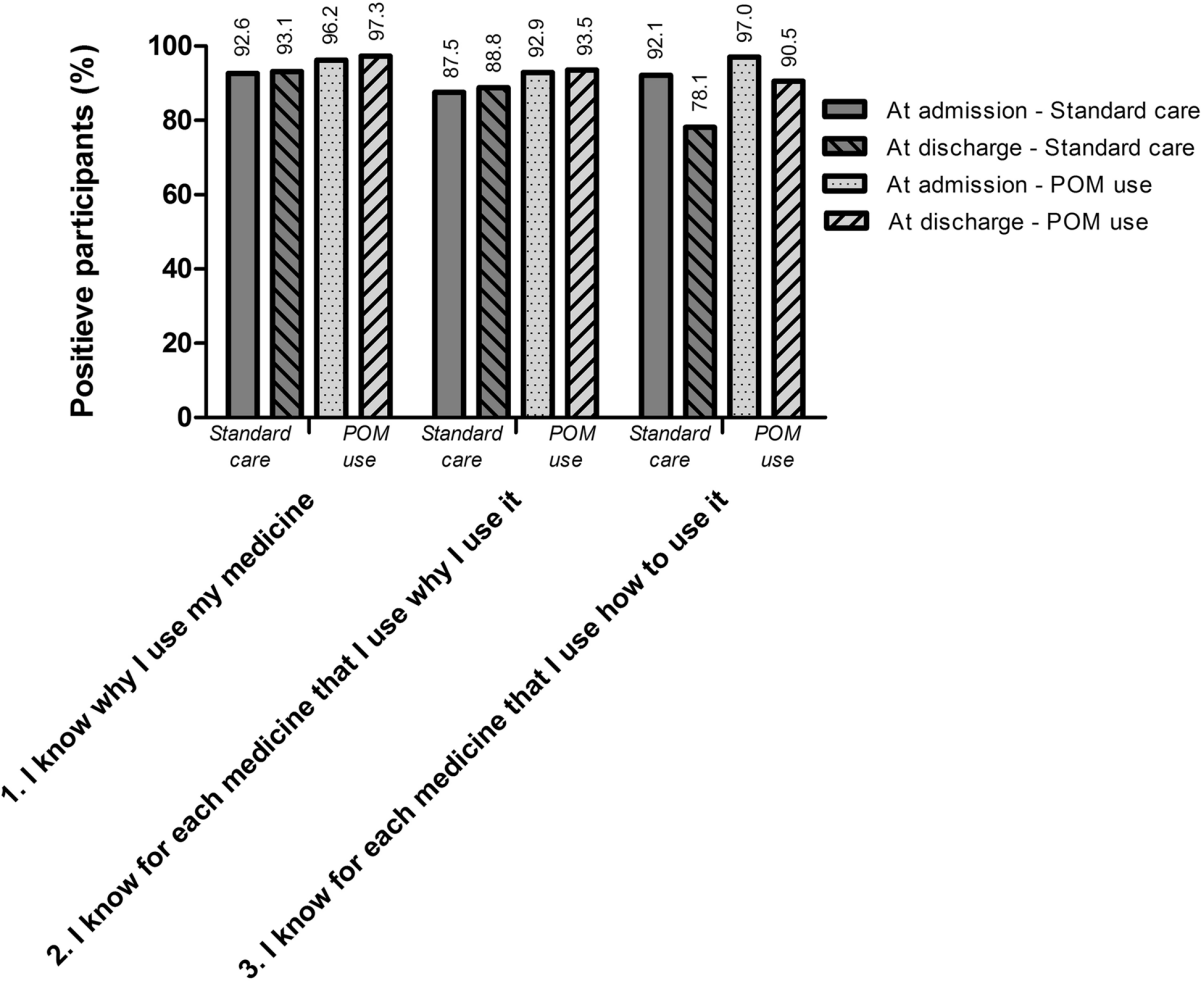
Proportion patients that reported to have adequate medication knowledge by patients receiving either standard care or POM use. POM = Patient’s Own Medication

The majority of the patients reported adequate medication knowledge during hospitalisation in both study periods, as shown in Fig. 1. At discharge, the proportion of patients who reported adequate medication knowledge about *why* they use their medicines (statement C1 and C2) was comparable between the SC (93.1% [C1] and 88.8% [C2]) and POM use (97.3% [C1] and 93.5% [C2]) group. Similar results were found at admission; adequate medication knowledge was reported in 92.6% (A1) and 87.5% (A2) of patients receiving SC and 96.2% (A2) and 92.9% (A2) of patients participating in POM use schemes, respectively. On the other hand, at discharge the proportion of patients with adequate self-reported medication knowledge on *how* to use medicines (C3) decreased from 92.1 to 78.1% in the SC group compared to 97.0 to 90.5% in the POM use group (Fig. 1).

In a multivariate model, POM use was significantly associated (adjusted OR (OR_adj_) [95% confidence interval (CI)]: 3.22 [95% CI 2.01–5.16]) with adequate medication knowledge at discharge on *how* to use medicines (C3). Furthermore, adequate medication knowledge at admission (C1: OR_adj_ 68.06 [95% CI 26.52–174.71], C2: OR_adj_ 41.16 [95% CI 19.87–85.26], C3: OR_adj_ 7.31 [95% CI 3.46–15.45]) and help with medication management at home (C1: OR_adj_ 0.35 [95% CI 0.12–0.97], C2: OR_adj_ 0.35 [95% CI 0.16–0.74], C3: OR_adj_ 0.36 [95% CI 0.21–0.59]) were significantly associated with adequate medication knowledge at discharge for all three statements (see Table 2. Multivariate logistic regression results of self-reported adequate medication knowledge at discharge.

**Table 2:** Multivariate logistic regression results of self-reported adequate medication knowledge at discharge

Statement	Self-reported medication knowledge at discharge			
**C1. I know** ***why*** **I use my medicines**	**Adequate n (%)** **695 (100)**	**Inadequate n (%)** **36 (100)**	**OR**_**crude**_ [95%CI]*	**OR**_**Adjusted**_ [95%CI]*
POM use				
No	366 (52.7)	27 (75.0)	Ref.	Ref.
Yes	329 (47.3)	9 (25.0)	2.70 [1.25–5.82]	1.88 [0.70–5.01]
Self-reported medication knowledge at admission (A1. I know *why* I use my medicines)				
Inadequate (negative or neutral answer)	20 (2.9)	22 (61.1)	Ref.	Ref.
Adequate (positive answer)	675 (97.1)	14 (38.9)	53.04 [23.73–118.53]	68.06 [26.52–174.71]
Gender^#^				
Male	388 (55.8)	21 (58.3)	Ref	Ref.
Female	298 (42.9)	15 (41.7)	1.08 [0.54–2.12]	0.72 [0.28–1.86]
Help with medication management at home^^^				
No	491 (70.6)	19 (52.8)	Ref.	Ref.
Yes	99 (14.2)	11 (30.6)	0.35 [0.16–0.76]	0.35 [0.12–0.97]
Native				
No	38 (5.5)	2 (5.6)	Ref.	Ref.
Yes	657 (94.5)	34 (94.4)	1.02 [0.24–4.39]	1.51 [0.23–9.82]
**C2. I know for each medicine that I use*why*I use it**	**Adequate n (%)** **665 (100)**	**Inadequate n (%)** **66 (100)**	**OR**_**crude**_ [95%CI]*	**OR**_**Adjusted**_ [95%CI*]
POM use				
No	349 (52.5)	22 (33.3)	Ref.	Ref.
Yes	316 (47.5)	44 (66.7)	1.81 [1.06–3.09	1.33 [0.67–2.65]
Self-reported medication knowledge at admission (A2. I know for each medicine that I use *why* I use it)				
Inadequate (negative or neutral answer)	30 (4.5)	43 (65.2)	Ref.	Ref.
Adequate (positive answer)	635 (95.5)	23 (34.8)	39.57 [21.19–73.92]	41.16 [19.87–85.26]
Gender^#^				
Male	372 (55.9)	37 (56.1)	Ref.	Ref.
Female	285 (42.9)	28 (42.4)	1.01 [0.61–1.69]	0.51 [0.25–1.03]
Help with medication management at home^^^				
No	470 (70.7)	40 (60.6)	Ref.	Ref.
Yes	90 (13.5)	20 (30.3)	0.38 [0.21–0.69]	0.35 [0.16–0.74]
Native				
No	30 (4.5)	10 (15.2)	Ref.	Ref.
Yes	635 (95.5)	56 (84.8)	3.78 [1.76–8.13]	4.49 [1.46–13.76]
**C3. I know for each medicine that I use*how*to use it**	**Adequate n (%)** **613 (100)**	**Inadequate n (%)** **118 (100)**	**OR**_**crude**_ [95%CI]*	**OR**_**Adjusted**_ [95%CI*]
POM use				
No	307 (50.1)	86 (72.9)	Ref.	Ref.
Yes	306 (49.9)	32 (27.1)	2.68 [1.73–4.14]	3.22 [2.01–5.16]
Self-reported medication knowledge at admission (A3. I know for each medicine that I use *how* to use it)				
Inadequate (negative or neutral answer)	17 (2.8)	24 (20.3)	Ref.	Ref.
Adequate (positive answer)	596 (97.2)	94 (79.7)	8.95 [4.63–17.29]	7.31 [3.46–15.45]
Gender^#^				
Male	350 (57.1)	59 (50.0)	Ref.	Ref.
Female	255 (41.6)	58 (49.2)	0.74 [0.50–1.10]	0.57 [0.36–0.90]
Help with medication management at home^^^				
No	435 (71.0)	75 (63.6)	Ref.	Ref.
Yes	73 (11.9)	37 (31.4	0.34 [0.21–0.54]	0.36 [0.21–0.59]
Native				
No	33 (5.4)	7 (5.9)	Ref.	Ref.
Yes	580 (94.6)	111 (94.1)	1.11 [0.48–2.57]	0.43 [0.14–1.31]

*Used aberrations*: *POM* Patient’s Own Medication, *OR* Odds Ratio, *CI* Confidence interval

~ Enter method; # 9 cases missing; ^ 111 cases missing

Being native Dutch was associated with a positive outcome for statement C2 “I know for each medicine that I use *why* I use it” (OR_adj_ 4.49 [95% CI 1.46–13.76]) and being female was associated with a negative outcome for statement C3 “I know for each medicine that I use *how* to use it” (OR_adj_ 0.57 [95% CI 0.36–0.90]).

### Secondary outcomes

#### Sense of medication safety

More than half of the patients perceived that POM use reduces the number of medication errors (Table 3, B1). This percentage was highest among POM users compared to SC patients (74.0% vs 52.9%; *P* <  0.01).

**Table 3:** Proportion patients that is positive towards a statement during standard care versus POM use

Statement (part B)	Moment of response	Standard care (% positive)	POM use (% positive)	***P*** value*
Sense of medication safety				
B1 I think that continued use of the medicines I use at home reduces the number of medication errors	At discharge	52.9	74.0	< 0.01
Attitude towards the provision of information				
B2 During hospitalisation it is clear to me which medicines from home I still use.	During hospitalisation	85.8	92.3	0.01
B3 I am informed about replacing a medicine that I use at home with a medicine from the hospital.	During hospitalisation	65.4	67.8	0.50
B4 When medication is started during the admission, I am informed about this.	During hospitalisation	76.3	79.6	0.29
B5 I have no more questions about my medication after admission.	At discharge	85.2	89.3	0.10
B6 I know where or to whom I can go with my questions about medication.	At discharge	87.5	92.6	0.02
Attitude towards inpatient medication use				
B7 I would like to be able to continue to use my medicines from home during the admission.	At discharge	68.2	82.2	< 0.01
B8 I would like to manage my medicines from home (when they are used during the admission) by myself.	At discharge	56.2	62.7	0.08

*Used aberrations*: *POM* Patient’s Own Medication

* Pearson Chi-Square test

#### Attitude towards the provision of information

In general most patients were positive towards the provision of information as shown in Table 3 (B2–6). Significantly more POM users (92.3%) reported to know which medicines from home they were still using during admission compared to patients receiving SC (85.8%; *P* <  0.01). In both groups, a comparable proportion of patients reported to be informed about substituted medication and newly started pharmacotherapy during hospitalisation. At discharge, similar proportions of patients reported to have no more questions. However, POM users knew better where to go with questions at discharge compared to SC (92.6% vs 87.5%; *P* = 0.02).

#### Attitude towards inpatient medication use

A significantly greater proportion of POM users would like to be able to use POM during hospitalisation, compared to SC patients (82.2% vs 68.2%; *P* < 0.01; Table 3 B7). The majority of the patients would also like to self-manage their medication use during hospitalisation, 56.2 and 62.7% (*P* = 0.08; Table 3 B8) in the SC and POM use group respectively.

## Discussion

In this study we explored the impact of POM use on self-reported medication knowledge at patient’s discharge from the hospital. The results showed that POM use seemed to be positively associated with patient’s knowledge about *how* to use medication at discharge. However, for the two other knowledge related questions no effect of POM use was observed, as the effect at discharge was highly determined by the patients’ perceived knowledge at admission. Furthermore, most patients perceived that POM use reduces the number of medication errors. POM use during hospitalisation seems to have a positive but small effect on being informed as patients reported to know better where to indicate questions at discharge. Moreover, most patients would like to be able to use POM during hospitalisation regardless of having experienced it. Therefore POM use seems a valuable intervention.

In this study, multiple variables seem to be associated with adequate self-reported medication knowledge at discharge. Notable is the high percentage of adequate self-reported medication knowledge at admission in both groups. These findings probably resulted into the high observed ORs with wide 95% CI, which may limit the clinical interpretation of the results. Despite the high percentage of baseline self-reported medication knowledge we did found a positive association between medication knowledge about the usage of medication at discharge and POM use. Recognizing the medication (packages) used during hospitalisation may account for this.

At admission we observed a significant difference in medication knowledge and medication use favouring POM users. This might be explained by the lower proportion of patients getting help with medication management at home in the POM use group. Patients who received no help might have a more positive attitude towards their medication knowledge and use. Furthermore, patients with better medication knowledge might be more likely to participate in POM use schemes during hospitalisation.

In our study, patients perceived that medication safety during hospitalisation would be improved by POM use. This may be because the patient is the last link in the medication process before administration and therefore could be the last one to correct a medication error before it actually reaches the patient. POM use may increase patient’s involvement which in turn could positively influence patient’s alertness resulting in patients feeling safer. This theory is supported by our study results as POM users were even more positive towards medication safety due to POM than patients receiving SC. Furthermore, results of previous research showed that patients believe that active participation during hospitalisation might prevent medication errors [18]. Although actual medication safety was not investigated in our study the feeling of being safe is relevant, as feeling unsafe can result in patient distress [23].

The majority of patients would like to participate in POM use schemes during hospitalisation and manage their medication by themselves (e.g. self-administration during hospitalisation), independent of experiencing it during this study. Moreover POM users were even more positive than patients receiving SC. Thus patients prefer to manage their own pharmaceutical care regardless of being hospitalised. Previous research showed that patients may benefit from self-administration [24–26]. An explanation for our findings could be that POM use may enhance patient counselling during hospitalisation. Indeed, previous research showed that patients prefer to receive medication information during hospitalisation rather than from community health care services [27]. Our findings may suggest that more information was provided or asked for during the POM use period because more patients reported to know which medicines from home they still used during hospitalisation and where or to whom they could go with medication questions. From patients’ perspectives we therefore recommend to implement POM use during hospitalisation.

The results of this study are representative for the entire Dutch hospital setting, due to the large number of patients included and the diversity among included hospitals and wards. However, more attention is necessary to include non-natives to use POM as language and culture may influence willingness to use POM. In addition, the standard medication process might differ in other countries when compared to our setting. Consequently, our findings may not be generalizable to other settings. Nevertheless, an important reason to implement POM is to make the medication recognizable for patients. As in many countries medication is currently substituted at admission we think that implementing POM use is a promising way to improve patient knowledge on medication use in other healthcare systems as well [28]. Moreover, there is a global trend to improve medication safety throughout the care setting and POM may contribute to this as less medication changes during transition of care decrease the opportunity for error.

This study had some limitations. First, almost 20% of the questionnaires could not be used for analysis because data were missing. This could have led to information bias. However, this exclusion was similar in both study groups making it plausible that this effect would be comparable. Second, no information on medication schedule was collected in this study, so we did not know how much and which medication patients used. Pharmacotherapeutic adjustments (started, stopped or changed medication) during hospitalisation were not collected either. We did not collect this (and other) information due to the fact that the study was designed to implement POM as standard practice. Therefore, we wanted to simulate normal conditions on the medical wards, with limited presence of a researcher during the observation periods. Furthermore, we wanted to minimize the burden for healthcare professionals on the ward. It is plausible that a higher number of medicines used and/or more changes would have a negative effect on medication knowledge because it enlarges the amount and complexity of knowledge to process and reproduce. Moreover, specific medication groups could be more difficult to use which might impact medication knowledge as well. As this study design may have resulted into selection bias, we assume this effect was present in both study groups. Third, in this study the assessment of medication knowledge was self-reported by patients and not measured (by verifying patients’ understanding). Patients may have overestimated their medication knowledge as previous research in elderly patients confirmed [27]. Nonetheless, we were interested in perceived medication knowledge because it helps us understand patients’ view. Fourth, in our study the follow-up period was short, only during hospitalisation. A follow-up study would be valuable in order to establish whether the self-perceived knowledge persists after hospitalisation. Fifth, our study design was exploratory therefore *p*-values should be interpreted with caution. Sixth, the answers ‘unknown’ and ‘not applicable’ were included in the negative/neutral response group. Therefore the group of patients with inadequate knowledge may be overestimated. However, as these answers were rarely given (only in 0–2% of responses), the overestimation will be limited. Last, literature could not provide us with an applicable, validated questionnaire. Therefore an expert team developed a questionnaire based on validated questionnaires [21, 22].

In conclusion, POM use has the ability to positively influence patient’s medication knowledge about *how* to use medication at discharge. Furthermore, POM use enhances the feeling of medication safety during hospitalisation and more patients have a positive attitude towards the provision of information due to POM use. Moreover, most patients prefer POM use during hospitalisation and would like to self-administer medication as well. Therefore, POM use seems a valuable intervention and more research towards POM use (in combination with self-administration) is recommended.

## Supplementary Information

**Additional file 1.**

## Data Availability

The datasets used and/or analysed during the current study are available from the corresponding author on reasonable request.

## Abbreviations

POM: Patient’s Own Medication
SC: Standard care
CI: Confidence interval
