# Supplementary material for: The effect of Patient’s Own Medication use on patient’s self-reported medication knowledge during hospitalisation: a pre-post intervention study
Source: BMC Health Serv Res. 2022 Mar 30;22:423. doi: 10.1186/s12913-022-07752-6 (PMC8969375; doi:10.1186/s12913-022-07752-6)
Supplement: Supplementary file 1 — Additional file 1. [file 12913_2022_7752_MOESM1_ESM.pdf]

The effect of Patient's Own Medication use on patient's self-reported medication knowledge during hospitalisation: a pre-post intervention study

Authors

Loes J.M. van Herpen-Meeuwissen[1,2], Bart J.F. van den Bemt[1,3,4], Hieronymus J. Derijks[1,5], Patricia M.L.A. van den Bemt[6], Barbara Maat[2], Hein A.W. van Onzenoort[1,4]

Patient questionnaire with statements on perceived medication knowledge, medication safety, the provision of information, and inpatient medication use.

**Date**	. . / . . / . . . .
**Gender**	Male – Female
**Year of birth**	. . . .
**Educational level**	◻ Elementary school<br>◻ Lower secondary education<br>◻ Upper secondary school<br>◻ Bachelor degree or higher<br>◻ Unknown
**Marital status**	◻ Single<br>◻ Partner, not married<br>◻ Married<br>◻ Divorced<br>◻ Widow/widower<br>◻ Unknown
**Do you receive help with medication management at home?**	Yes – No
**What is your first language**	. . . .

Please response to the statements about medication use	Totally disagree	Disagree	Neutral	Agree	Totally agree
01. I always take my prescribed medicine					
02. I always take the prescribed amount of medicine					
03. I always use my medicine at the prescribed time					

Please response to the statements below at your hospital admission.

**A. Medication Knowledge (at admission)**	Totally disagree	Disagree	Neutral	Agree	Totally agree
A1. I know *why* I use my medicines					
A2. I know for each medicine that I use *why* I use it					
A3. I know for each medicine that I use *how* to use it					

Please response to the statements below during your hospitalisation.

**B. Sense of medication safety, provision of information, and inpatient medication (during hospitalisation)**	Totally disagree	Disagree	Neutral	Agree	Totally agree
B1. I think that continued use of the medicines I use at home reduces the number of medication errors.					
B2. During hospitalisation it is clear to me which medicines from home I still use					
B3. I am informed about replacing a medicine that I use at home with a medicine from the hospital.					
B4. When medication is started during admission, I am informed about this.					
B5. I would like to be able to continue to use my medicines from home during the admission.					
B6. I would like to manage my medicines from home (when they are used during the admission) by myself.					

Please response to the statements below at your hospital discharge.

**C. Medication Knowledge and provision of information (at discharge)**	Totally disagree	Disagree	Neutral	Agree	Totally agree
C1. I know *why* I use my medicines					
C2. I know for each medicine that I use *why* I use it					
C3. I know for each medicine that I use *how* to use it					
C4. I have no more questions about my medication after admission.					
C5. I know where or to whom I can go with my questions about medication.					
